# The Increase of Theta Power and Decrease of Alpha/Theta Ratio as a Manifestation of Cognitive Impairment in Parkinson’s Disease

**DOI:** 10.3390/jcm12041569

**Published:** 2023-02-16

**Authors:** Katarzyna Zawiślak-Fornagiel, Daniel Ledwoń, Monika Bugdol, Patrycja Romaniszyn-Kania, Andrzej Małecki, Agnieszka Gorzkowska, Andrzej W. Mitas

**Affiliations:** 1 Department of Neurology, University Clinical Center Prof. K. Gibiński, Medical University of Silesia, 40-752 Katowice, Poland; 2 Faculty of Biomedical Engineering, Silesian University of Technology, 41-800 Zabrze, Poland; 3 Institute of Physiotherapy and Health Sciences, The Jerzy Kukuczka Academy of Physical Education in Katowice, 40-065 Katowice, Poland; 4 Department of Neurorehabilitation, Faculty of Medical Sciences in Katowice, Medical University of Silesia, 40-752 Katowice, Poland

**Keywords:** Parkinson’s disease, dementia, mild cognitive impairment, quantitative EEG, spectral analysis

## Abstract

In this study, we aim to assess and examine cognitive functions in Parkinson’s Disease patients using EEG recordings, with a central focus on characteristics associated with a cognitive decline. Based on neuropsychological evaluation using Mini-Mental State Examination, Montreal Cognitive Assessment, and Addenbrooke’s Cognitive Examination-III, 98 participants were divided into three cognitive groups. All the particpants of the study underwent EEG recordings with spectral analysis. The results revealed an increase in the absolute theta power in patients with Parkinson’s disease dementia (PD-D) compared to cognitively normal status (PD-CogN, p=0.00997) and a decrease in global relative beta power in PD-D compared to PD-CogN (p=0.0413). An increase in theta relative power in the left temporal region (p=0.0262), left occipital region (p=0.0109), and right occipital region (p=0.0221) were observed in PD-D compared to PD-N. The global alpha/theta ratio and global power spectral ratio significantly decreased in PD-D compared to PD-N (*p* = 0.001). In conclusion, the increase in relative theta power and the decrease in relative beta power are characteristic changes in EEG recordings in PD patients with cognitive impairment. Identifying these changes can be a useful biomarker and a complementary tool in the neuropsychological diagnosis of cognitive impairment in Parkinson’s Disease.

## 1. Introduction

Parkinson’s disease (PD) is a progressive degenerative disease of the nervous system. It is the fastest-growing neurological disease in terms of prevalence and associated disability [1]. PD affects more than 1% of the population over 65 years old, with a prevalence predicted to double by 2030 [2]. The disease is caused by a deficiency of neurotransmitters, mainly dopamine, due to the death of substantia nigra cells. The main symptoms of PD include abnormalities of movement, such as tremors, slow movement, and rigidity. The International Parkinson and Movement Disorder Society (MDS) defines PD as the presence of bradykinesia combined with either resting tremor, rigidity, or both [3]. No less important but often overlooked are nonmotor symptoms such as autonomic disorders, neuropsychiatric disorders, sleep disorders, and others, for example, pain, diplopia, or olfactory disorders [4]. Cognitive impairment is the most frequent and disabling nonmotor disorder among them. It is the patient’s mental disability that aggravates the course of the disease and the quality of life for PD patients [5,6]. Furthermore, it has a significant negative impact on the patient’s caregiver, becoming an additional burden over time [7]. Cognitive impairment increases health-related costs, the risk of nursing home admission, and the average duration of hospital visits [8,9]. The timing, profile, and rate of cognitive decline vary notably among individuals with PD. It can range from normal cognition (PD-CogN) to mild cognitive impairment (PD-MCI) and dementia (PD-D) [10]. It is estimated that an average of 26.7% (range 18.9–38.2%) of PD patients without dementia have mild cognitive impairment [11]. The most important factors triggering the development of cognitive decline in PD are the duration of the disease, advanced age, the severity of movement disorders, akinetic-rigid form, and the presence of pre-existing mild cognitive impairment [12].

The prevalence of dementia in individuals with PD is estimated to be approximately 30%, with an incidence rate that is up to six times higher when compared to non-PD patients. The presence of PD-MCI has been found to significantly increase the risk of progression to dementia, with studies indicating a sixfold increase in likelihood [12]. In the matter of psychological examination in PD patients, the domains usually damaged by the disease should be taken into account—visual and spatial disorders, verbal fluency disorders, abstract thinking, and planning disorders being dominant in such cases [13]. Recent research has focused on the identification of cognitive decline in PD at an early stage in order to provide therapeutic interventions or to target individuals at high risk of dementia. This is aimed at slowing down the progression of cognitive decline and improving the quality of life for patients [14,15]. Consequently, the ongoing pursuit of effective diagnostic tools is a crucial area of research in this field of medicine. The discovery of biomarkers that can accurately identify individuals with cognitive deterioration in PD would be of significant value for the investigation of disease progression and the implementation of potential prevention at an early stage [16,17]. This would be particularly useful when applied to the management of cognitive decline in PD patients.

Following the example of biomarkers in Alzheimer’s disease, the researchers have examined and analyzed changes in the genetic background, cerebrospinal fluid, other biological fluids, and microstructural brain changes in individuals with PD [18,19]. Despite advanced research techniques and continuous yet unsuccessful attempts to establish an accurate marker of cognitive disorders in PD, they continue to be difficult to diagnose.

Research shows that electroencephalography appears to be a promising diagnostic tool for cognitive disorders. Electroencephalography (EEG) is a noninvasive technique that can depict electric brain activity in a high temporal resolution. In the context of PD, it requires minimal patient cooperation and is independent of motor processing [20]. Quantitative EEG (QEEG) is an increasingly used form of modern EEG analysis. It involves recording digital EEG signals, which are later processed, transformed, and analyzed using complex mathematical algorithms. QEEG enables the extraction of the following characteristics of EEG signals: analysis of a specific frequency band, signal complexity, connectivity, and network analysis [21].

Currently, there are many approaches using EEG and QEEG parameters in statistical and machine learning methods to aid diagnosis. Due to the complexity of the mechanisms underlying some diseases, they are a significant diagnostic challenge. Previous works demonstrate the feasibility of using machine learning methods in the identification of many specific disorders, i.e., heart diseases [22,23], ischemic stroke [24], and seizure detection and prediction in epilepsy [25]. It was found that the QEEG parameters enable the creation of a healthcare assistance system for prognostics of ischemic stroke [24]. It can also provide neurological outcomes and poststroke rehabilitation management and could be helpful for poststroke treatment and poststroke recovery [26]. Moreover, the use of wearable EEG devices makes it possible to develop approaches in predicting neurological status used in advanced driver assistance [27] and sleep monitoring systems [28].

The use of qEEG parameters in the diagnosis of cognitive disorders has been addressed in several studies [29,30,31,32]. The results in this field have yet to lead to any general conclusions. There is no common opinion regarding which specific markers can be used to predict cognitive decline in PD. Due to rapidly developing methods and approaches, researchers investigate different methods. In addition, studies in this field have been conducted on relatively small groups. They can produce false positive results or overestimate the importance of correlations. In this case, a thorough comparison of QEEG patterns becomes a challenge.

This study focuses on exploring individual frequency bands of the EEG signal and their changes depending on the severity of cognitive disorders in PD. The study group includes patients with PD-MCI and PD-D, compared against a control group of PD patients without cognitive decline. The main aim of the research is to identify the characteristics within the spectral analysis of the EEG indicative of an early stage of cognitive impairment. Additionally, we investigated whether there is any correlation between age, disease duration, and the severity of the neurological state. Considered are The Unified Parkinson Disease Rating Scale (MDS-UPDRS) and Hoehn and Yahr Scale (H&Y), Levodopa Equivalent Dose (LED), and the observed changes in QEEG among the individual groups of patients. The key contributions of this paper can be summarized as follows:

- The extraction of QEEG coefficients using modern equipment and tools was conducted.
- The results of a relatively large group of patients with Parkinson’s disease who underwent comprehensive neuropsychological assessments were included.
- Data has been collected during routine diagnostics. Thus, the results were collected in real conditions, not only to assemble a group for research.
- Statistical analysis was conducted to identify biomarkers of MCI and dementia.

## 2. Materials and Methods

### 2.1. Subjects

**Figure 1:**
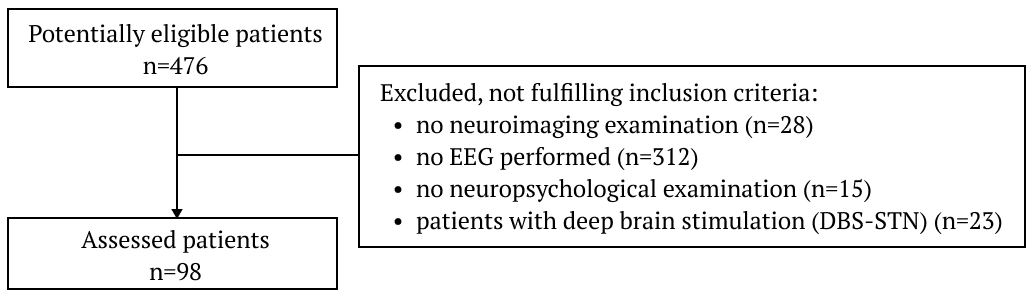
Flow chart of patients selection.

The research group was recruited from the Neurology Ward and the Single-Day Ward of the Neurology Clinic of the Silesian Medical University in Katowice between the 1 August 2019 and the 30 October 2022. The research was carried out retrospectively based on the medical data analysis and EEG recordings performed during the diagnostic process. The study was conducted according to the guidelines of the Declaration of Helsinki. Ethical review and approval were waived for this study due to the retrospective character of the work and data anonymization. The Ethics Committee of the Medical University of Silesia waived the requirement to obtain ethical approval for this study. The patients with PD were diagnosed according to the Movement Disorder Society clinical diagnostic criteria for Parkinson’s disease 2015 [3]. The diagnosis of PD-MCI was performed in line with the Movement Disorder Society Task Force Guidelines 2012 [11], and the PD-D was made according to Clinical Diagnostic Criteria for Dementia associated with Parkinson’s disease formed by MDS Task Force in 2007 [12]. A total of 453 patients with PD were hospitalized at the chosen time. To be included in the study, patients must have met the aforementioned disease criteria: undergo a brain neuroimaging examination (computer tomography or MRI of the brain), undergo an EEG examination, and have a neuropsychological consultation. Exclusion criteria included: atypical and secondary parkinsonism, implantation of deep brain stimulation, the presence of other neurological or psychiatric conditions, other secondary causes of cognitive impairment that were significant according to the evaluator (e.g., decompensated, advanced hypothyroidism, significant electrolyte disturbance), and any other severe illnesses. Figure 1 depicts the participants’ recruitment flow chart. Based on the neuropsychological examination and the currently applicable PD-D and PD-MCI diagnosis criteria, 25 patients with PD-D, 30 patients with PD-MCI, and 43 patients without cognitive impairment were selected for the study. Detailed data of the clinical characterization are summarized in Table 1.

### 2.2. Clinical and Neuropsychological Assessment

After collecting a thorough medical history, all patients underwent a comprehensive neurological examination. The interview was conducted with the patients and with their family members present for precise data collection, inclusive of demographics as well as clinical and pharmacological data. The severity of PD was evaluated with MDS-UPDRS and H&Y scales. Patients were examined in both ‘OFF’ and ‘ON’ motor states—before and after taking an appropriate dose of Levodopa. The daily dosage of dopaminergic drugs was converted using the Levodopa Equivalent Dose (LED).

Patients were examined using a series of standardized neuropsychological diagnostics, assessing the general level of cognition and the individual cognitive domains. This includes memory, attention, language, visuospatial function, and executive function. The assessment was carried out in the “ON” motor state, with a composite battery of tests, including the Mini-Mental State Examination (MMSE), Montreal Cognitive Assessment (MoCA), clock drawing test (CDT), Addenbrooke’s Cognitive Examination III (ACE III), Benton Visual Retention Test (BVRT). Behavioral assessment was made according to Beck Depression Inventory (BDI). Other causes of cognitive impairment were excluded based on the neuroimaging and diagnostic laboratory testing.

### 2.3. Eeg Recording

All the EEG recordings were obtained from PD patients in the “ON” state during routine diagnostics using the EEG device with an 87-channel Natus Brain Monitor amplifier from 19 electrodes positioned according to the 10–20 International System (Fp1, Fp2, F3, F4, F7, F8, Fz, C3, C4, Cz, T3, T4, T5, T6, P3, P4, Pz, O1, O2). EEG activity was analyzed from single or multiple leads, grouped to define the following scalp regions: frontal (Fp1, Fp2, F3, F4, Fz), central (C3, C4, Cz), temporal (F7, F8, T3, T4, T5, T6), parietal (P3, P4, Pz) and occipital (O1, O2). Data was recorded with the sampling frequency 512 Hz. All records were done in a resting, awake condition with the eyes closed. The duration of the entire routine EEG examination was 20 min. The patients were instructed to relax and stay awake, minimizing eye and body movements. The EEG technician monitored all subjects during the recordings to keep watch over the vigilance and artifacts. None of the patients took medications that might influence the EEG recording (antiepileptic or antipsychotic drugs).

### 2.4. Processing of Eeg Data

The EEG recordings were analyzed with the MNE-Python framework [33]. Signals were firstly filtered with a notch 50 Hz filter to reduce power line noise and a high-pass FIR 0.1 Hz filter with Hamming window. Next, each EEG signal was split into 5-s epochs. For each epoch, we used baseline correction and detrending. Next, based on the technician’s annotation, we removed epochs with unexpected events which impacted the signal (i.e., movement, blinking, speaking). Other epochs with artifacts were removed according to the Autoreject method proposed by Jas et al. [34]. The resulting epochs were manually verified by an expert for the further presence of artifacts.

The standard EEG test protocol includes different conditions during the recording (i.a., stroboscope and hyperventilation tests). In this study, we selected epochs from initial fragments with resting state and eyes closed conditions (approximately 3 min long). Each epoch was re-referenced to the average of all channels. The QEEG parameters were extracted based on the power spectral density (PSD) computed with the multitaper method from 0.5 to 45 Hz [35]. The PSD signals from all epochs were averaged to receive a single PSD for each channel for one patient. The absolute and relative power in each electrode and for the average PSD was calculated in different frequency bands: delta (0.5–4 Hz), theta (4–8 Hz), alpha (8–13 Hz), low alpha (8–10 Hz), high alpha (10–13 Hz), beta (13–30 Hz) and gamma (30–45 Hz). We also computed two different power ratios: alpha/theta ratio [36] and Spectral Power Ratio (SPR) (alpha + beta)/(delta + theta) [37]. To perform the connectivity analysis, we used the Phase Lag Index (PLI) measure. The global PLI value for theta, alpha, beta, and gamma waves was determined by averaging the PLI from all connections between channels. PLI values between the defined regions were also determined by averaging the connectivity values between the electrodes in the analyzed regions.

### 2.5. Statistics

Statistical comparison of the three groups has been preceded by verifying the distribution normality (Shapiro–Wilk test) as well as sample size equality (chi-square goodness of fit test). In case of violating those assumptions, the Kruskal–Wallis test has been computed with the pairwise Wilcox test for unpaired samples with Holm’s correction. The effect size has been evaluated using epsilon squared. When normality and equinumerosity were fulfilled, the variance homogeneity was checked (Levene’s test). Depending on the obtained test result, ANOVA or ANOVA with Welch’s correction was performed. The effect size was estimated using eta squared, and the post-hoc analysis was computed with the pairwise *t*-test for unpaired samples or pairwise Welch’s test, respectively, both with Holm’s correction. Correlation analysis was calculated using Pearson’s correlation coefficient (for two variables on the interval scale) and Spearman’s correlation coefficient (if at least one of the variables was on the ordinal scale). The significance level was set to α=0.05.

## 3. Results

### 3.1. Cohort Clinical Characteristics

The clinical characteristics are presented in Table 1. The statistical analysis showed significant differences between the groups. The PD-MCI and PD-D patients were older than PD-CogN (*p* = 0.0137 and *p* = 0.0003, respectively). In the investigated population, PD-D patients had a significantly longer duration of the disease (*p* = 0.0007) as well as greater disease severity assessed by MDS-UPDRS III OFF (*p* = 0.0003) and Hoehn–Yahr stage (*p* = 0.0053) compared to PD-CogN patients. The median Levodopa equivalent dose was also statistically higher in PD-D relative to PD-CogN patients (*p* = 0.0093).

### 3.2. Qeeg Spectral Power Frequency Analysis

Global absolute theta power significantly differed between the three subgroups PD-D, PD-MCI, and PD-CogN (*p* = 0.0036; Table 2). In subgroup comparison, it was found that global absolute theta power increased for individuals with PD-MCI and PD-D compared to individuals with PD-CogN. However, a statistically significant difference was only observed between PD-D and PD-CogN (*p* = 0.0032 for absolute theta power). In terms of the remaining waveforms (absolute delta, alpha, and beta frequencies), no significant differences were observed.

**Figure 2:**
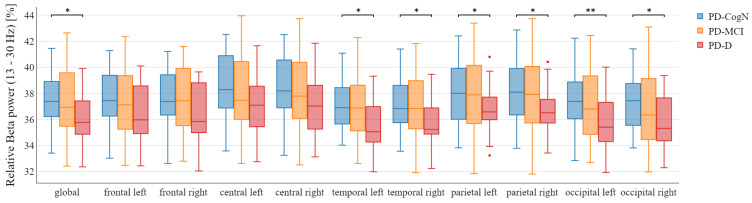
Relative beta power in the different areas between patient’s groups with statistical significance highlighted (* p<0.05, ** p<0.01).

**Figure 3:**
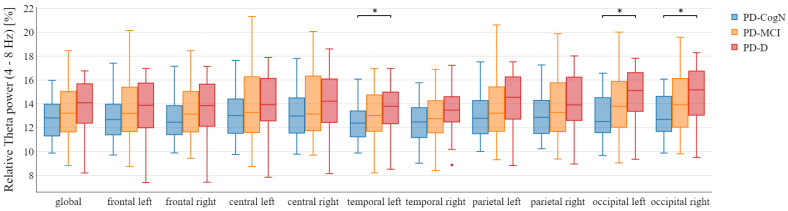
Relative theta power in the different areas between patient’s groups with statistical significance highlighted (* p<0.05).

The mean and standard deviation of global relative power in each band has been presented in Table 3. In the post hoc analysis, a significant decrease in the global relative beta power was observed in PD-D, compared to PD-N (*p* = 0.0374). Looking at the individual regions, the data analysis explores a significant decrease of relative beta power in the temporal, parietal, and occipital regions in PD-D, compared to PD-CogN (Figure 2). Moreover, the significant increase in theta relative power in the left temporal region (*p* = 0.0262), left occipital region (*p* = 0.0109), and right occipital region (*p* = 0.0221) in PD-D compared to PD-CogN (Figure 3).

**Figure 4:**
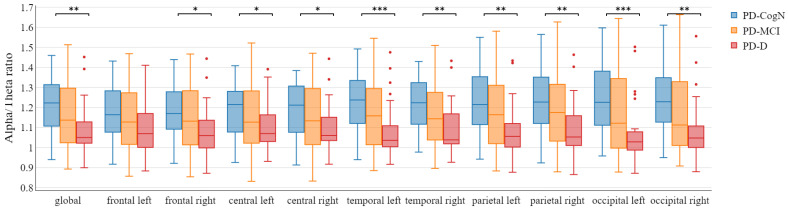
Alpha/theta ratio in the different areas between patient’s groups with statistical significance highlighted (* p<0.05, ** p<0.01, *** p<0.001).

**Figure 5:**
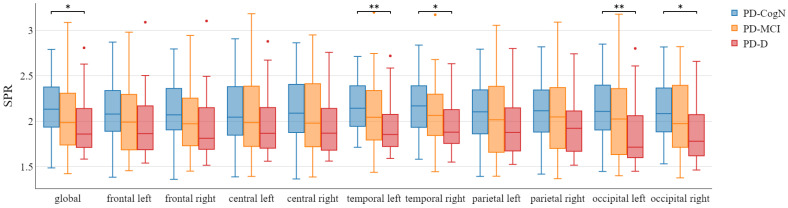
Spectral PowerRatio in the different areas between patient’s groups with statistical significance highlighted (* p<0.05, ** p<0.01).

**Figure 6:**
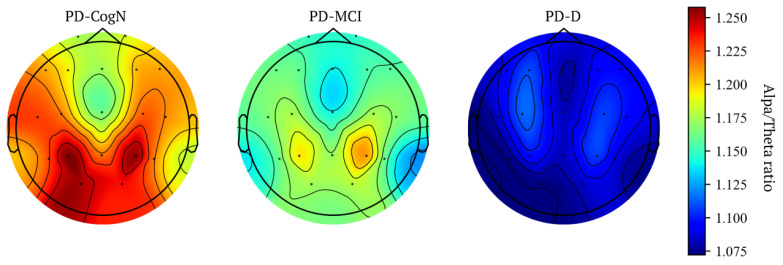
Average topographic maps of Alpha/Theta ratio in patient’s groups.

**Figure 7:**
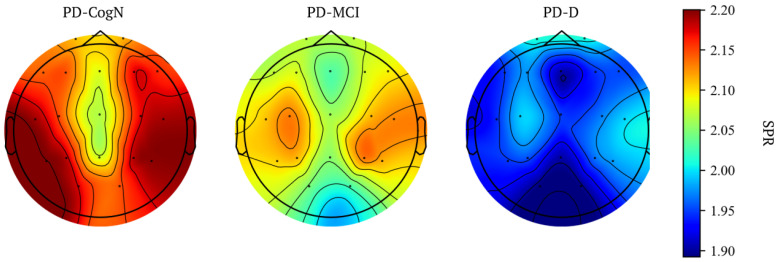
Average topographic maps of SPR in groups.

The global alpha/theta ratio and global SPR significantly differed between the three subgroups PD-D, PD-MCI, and PD-CogN (p=0.004) as seen in Table 3. Comparing the groups, a statistically significant decrease is observed in PD-D compared to PD-CogN (p=0.001). The alpha/theta ratio significantly differed between PD-D and PD-CogN in almost all regions of the brain (Figure 4). Whereas the SPR significantly differed (PD-D vs PD-CogN) in bilateral temporal and occipital regions (Figure 5). These results are also presented as average topographic maps of alpha/theta ratio and spectral power ratio in the studied subgroups (Figure 6 and Figure 7).

**Figure 8:**
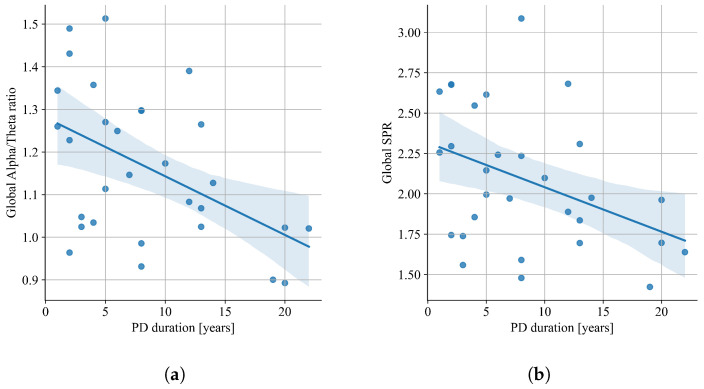
Linear regression of global alpha/theta ratio (**a**) and global SPR (**b**) versus PD duration in PD-MCI group.

**Figure 9:**
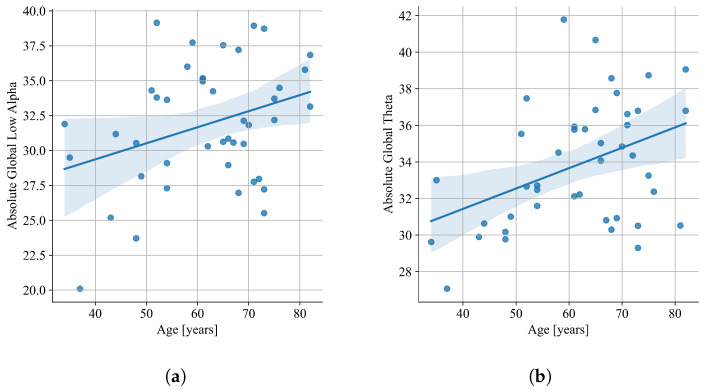
Linear regression of absolute global low alpha (**a**) and absolute global theta (**b**) versus age in PD-CogN group.

Correlation analysis performed on the results of all patients showed no correlation of spectral QEEG parameters with patients’ characteristics. Further analysis in separate groups of patients resulting from particular cognitive impairments showed a correlation between some QEEG parameters and the patients’ age and PD duration. The correlation analysis with Pearson’s coefficient showed a fair negative correlation of the global alpha/theta ratio and global SPR with PD-MCI patients’ disease duration (alpha/theta: r=−0.48, p=0.0069; SPR: r=−0.40, p=0.0296; Figure 8). In relation to the age of the patients, a fair positive correlation was shown for absolute global theta (r=0.41, p=0.021) and absolute global low alpha (r=0.33, p=0.031) (Figure 9). The analysis in separate groups showed no significant correlation of spectral QEEG parameters with other patients’ characteristics: MDS-UPDRS, H&Y, and LED.

**Figure 10:**
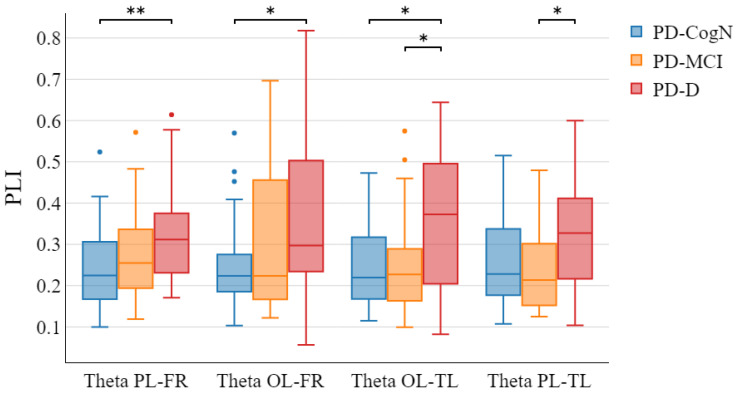
Distributions of theta PLI between selected regions which are significantly different between patient’s groups (* p<0.05, ** p<0.01).

The global PLI in theta, alpha, beta, and gamma waves showed no significant differences between groups. The functional connectivity analysis with PLI measure between regions showed statistically significant differences only in theta wave for specific areas (Figure 10). The post-hoc analysis revealed region connections in which PLI in the PD-D group were significantly higher than in PD-CogN: parietal left and frontal right (PL-FR; p=0.0039), occipital left and frontal right (OL-FR; p=0.0204), and occipital left and temporal left (OL-TL; p=0.0243). A significant PLI difference between the PD-MCI and PD-D groups was also observed for the two connections: occipital left and temporal left (OL-TL; p=0.0332) and parietal left and temporal left (PL-TL; p=0.0384).

## 4. Discussion

The research shows an increase in the global absolute theta power in PD-MCI compared to PD-N. Moreover, an increase in global absolute theta power was observed in PD-D compared to PD-MCI. However, a statistically significant difference was observed between PD-D and PD-N (p=0.00997). These findings are consistent with research done by Caviness et al. [29], which has examined the same study subgroups with a smaller sample size. Bousleimann et al. [32] based on the analysis of a high-resolution EEG proves a decrease in the low alpha power (8–10 Hz) in PD-MCI, compared to the PD-CogN. On the other hand, a nonsignificant association trend between theta power and PD-MCI was found in this study. The contrary results can be due to the small sample size used in the research as well as different diagnostic tools being used in assessing the cognitive state in the studies.

In their other study, Cavines et al. [38] examined the longitudinal changes in QEEG, analyzing the recordings and their correlation with cognitive impairments. It was found that the decreasing delta band power was the most likely marker of the MCI. This has been confirmed by further research, where an increasing trend in the delta band power was observed from PD-CogN to PD-MCI and to PDD [39]. A systematic review also concluded that PDD patients have increased amplitude in lower frequency bands, such as theta and delta, and decreased amplitude in higher frequency bands, alpha and beta [40]. It was observed that global EEG measures have potential use as biomarkers in the study of both early and late cognitive deterioration in PD [29].

There were no significant differences in absolute delta, alpha, and beta frequencies between the study groups in our study. However, analyzing the relative power of the spectrum, the study revealed statistical differences in the beta frequency band between groups. A significant decrease in global relative beta power was shown in PD-D, compared to PD-N (p=0.0374).

Looking at the individual regions, the data show an increase in the theta relative power in the left temporal region (p=0.0262), left occipital region (p=0.0109), and right occipital region (p=0.0221) in PD-D compared to PD-CogN. Similar results were obtained by He et al. [41], who examined a large group of 135 PD patients and 44 healthy members of the control group. They have discovered increasing theta frequency in the left posterior temporal region, left occipital region, and right frontal region among the PD-MCI patients compared to the control group. These findings have been confirmed by research showing that the high theta band power significantly increases the occurrence of PD-D. This leads to the conclusion that relative power in the theta band could be considered a potential biomarker of dementia in PD [42].

Moreover, our study shows that the relative beta powers decrease in PD-D compared to PD-N. It has been reported in the following areas: the bilateral temporal, bilateral parietal, and bilateral occipital regions. These findings are similar to He et al. [41] where they compared PD-MCI to the healthy control group, showing a decrease in relative beta powers in the bilateral posterior temporal, bilateral parietal, as well as left central regions. This was also confirmed by another study, where the beta band relative power decreased in PD and PD-MCI [29]. However, the high-resolution EEG study by Bousleimann et al. [32] showed a decrease of low alfa power in PD-MCI compared to PD-CogN (particularly in the right temporal region). Looking at these results, we agree with the claim that the global relative EEG power appears more useful in highlighting differences between the three investigated PD clinical cognitive states than the results from focal brain regions [29].

Significant indicators that establish the global slowing of the background activity are the alpha/theta ratio and spectral power ratio (SPR). In this study, the global alpha/theta ratio and global power spectral ratio significantly differed between the three subgroups PD-D, PD-MCI, and PD-CogN (p=0.004). It was observed to decrease, with increasing cognitive impairment PD-CogN > PD-MCI > PD-D. When comparing the groups, a statistically significant difference can be seen between PD-D and PD-CogN (p=0.001). The relative alpha/theta ratio significantly differs between PD-D and PD-CogN in almost all regions of the brain. The alfa/theta ratio in all regions is lower in PD patients than in the healthy control group, and it can be associated with visuospatial performance impairment in PD [43]. Another research study also indicates that the alpha/theta ratio is significantly decreasing in PD-D versus PD-MCI [31]. The study by Bousleimann et al. showed that the alpha/theta ratio could be a potential screening tool for mild cognitive impairment in PD patients [44].

In our results, the SPR significantly differs (PD-D vs. PD-N) in bilateral temporal and occipital regions. A similar result has been obtained in a recent study, in which power spectral ratios in frontal, central, temporal, parietal, and occipital regions were significantly decreased in PD patients compared to the healthy control group. Other researchers also confirm that SPR significantly decreased with a cognitive decline [45]. Several studies using a range of EEG and cognitive function measurements have also shown slowing in the power spectra associated with cognitive decline in PD [45,46,47,48,49]. The slowing in EEG also moderately correlates with MMSE scores [37]. Studies investigating the correlation between the slowing of the EEG in PD and cognitive impairment have proved that spectral power in a frequency range below 8 Hz was significantly increased in patients with dementia [30,50].

The slowing of background activity as an expression of brain disorganization in the course of the neurodegenerative process has been described by numerous researchers [29,30,47,48,51]. An increase in theta and delta activity is regarded to represent dysfunction in diffuse gray matter areas in both cortical and subcortical areas, as well as partial deafferentation of the cerebral cortex [52]. A decrease in background activity and the increase of low-frequency powers on QEEG correlate with cognitive impairment and have been suggested to be a consequence of cholinergic deficit [53]. Degeneration of the cholinergic system is an important factor for cognitive decline in PD [54]. More recently, research has shown that cholinergic degeneration may be particularly critical to early cognitive impairment in Parkinson’s disease [55,56].

Our study confirms, with the Pearson’s correlation coefficient, that the global alpha/theta ratio and global SPR are moderately linearly correlated with the disease duration in patients with PD-MCI (alpha/theta: r=−0.48, p=0.0069; SPR: r=−0.40, p=0.0296). Contrary to these results, no correlation between disease duration and relative theta powers between the subgroups of PD-MCI and PD (without MCI and healthy control groups) were found in similar studies [41]. However, they have found a positive correlation between the modified H&Y grading score and relative theta power in the left posterior temporal area [41]. Based on our analysis using the Pearson correlation, neither MDS-UPDRS, H&Y, and LED relate to the cognitive decline in PD, which is confirmed by Klassen et al. [42], where none of them account for the QEEG changes in PD cognitive decline. The changes in the theta and low alpha frequencies in patients with PD-N are moderately correlated to their age (relative theta power: r=0.37, p=0.0139; relative low alfa power r=0.38, p=0.0131). Low alfa power was also considered a useful marker for mild cognitive impairment [32].

Based on the functional connectivity analysis, it was shown that an increase in theta PLI between the occipital left and temporal left regions can be a promising biomarker of PD-D. It significantly differentiates PD-D from PD-MCI and PD-CogN. PLI theta patterns in the PD-D group were significantly higher than in the PD-CogN. This illustrates a higher degree of synchronization between these regions. Research shows that atrophy within the medial temporal lobe network correlates with progression to Parkinson’s disease dementia [56]. Using voxel-based morphometry MRI analysis, Pereira et al. showed that PD-MCI patients have greater grey matter atrophy in both occipitotemporal and dorsal parietal cortices compared to controls [57]. Baggio et al. revealed that the default mode network displayed increased connectivity with medial and lateral occipito-parietal regions in PD-MCI patients [58]. Altered connectivity between these regions may represent a risk factor for developing a clinically significant onset of an internalizing disorder and can be a marker of disease progression in PD [46]. There is also another hypothesis, in which a compensatory mechanism lies at the basis of these changes to maintain cognitive functions, as has been proven in the treatment of Alzheimer’s disease [59].

As shown in our study, QEEG may be useful in examining the cognitive decline in PD, complementing the detailed neuropsychological diagnosis. The results in the performed study display statistically significant differences between PD-D and PD-N groups. No similar tendencies have been noted between PD-MCI and PD-N. This can be due to the fact that PD-MCI is a highly varied group of individuals, including patients with close to standard cognitive abilities, as well as the ones with a high probability of progression to PD-D. The advantages and drawbacks of our research compared to other studies have been presented in Table 4. Our findings were supported by the relatively large sample size and comprehensive neurological assessments and the dataset from routine diagnostics.

## 5. Conclusions

1. Power spectral analysis of the PD-N, PD-MCI, and PD-D EEG recordings displays significant differences between the three groups.
2. The cognitive impairments in PD patients can be detected in the QEEG recordings through the following characteristics:
  (a) The slowing of background activity
  (b) The increase in relative theta power
  (c) The decrease in relative beta power
  (d) The decrease in the global alpha/theta ratio and global power spectral ratio
  (e) The increase in theta PLI in specific connections between the occipital left, temporal left, frontal right, and parietal left regions
3. QEEG analysis could become a useful and complementary diagnostic tool for the neuropsychological diagnosis of cognitive impairment in patients with Parkinson’s disease, but this still requires further research.

## Figures and Tables

**Table 1:** Clinical characteristics of PD-CogN, PD-MCI, and PD-D patients—mean (standard deviation).

	PD-CogN	PD-MCI	PD-D	*p*
N	43	30	25	-
Age (years)	61.79 (12.49)	68.27(6.20)	71.36 (5.34)	0.0002
PD duration (years)	8.65 (6.80)	8.40 (6.17)	13.88 (4.55)	0.0003
MDS-UPDRS III OFF	41.05 (14.82)	44.00 (18.85)	58.46 (15.65)	0.0003
Hoehn-Yahr stage	2.72 (0.80)	2.87 (0.86)	3.44 (0.87)	0.0057
LED (mg)	808.14(732.16)	845.07(532.14)	1291.88(685.37)	0.0082

**Table 2:** Mean (standard deviation) global absolute power in subgroups. Bold indicates statistically significant differences.

	PD-CogN	PD-MCI	PD-D	*p*
Delta (dB)	39.78 (2.73)	40.60 (3.52)	42.07(4.17)	0.0855
**Theta (dB)**	**33.86 (3.41)**	**35.62 (3.93)**	**37.03 (4.03)**	**0.0036**
Alpha (dB)	34.51 (4.00)	34.86 (3.30)	34.12 (3.14)	0.7501
Low Alpha (dB)	31.87 (4.36)	32.30 (3.53)	31.76 (3.43)	0.8539
High Alpha (dB)	30.58 (3.88)	30.77 (3.64)	30.19 (2.99)	0.8367
Beta (dB)	32.67 (3.08)	32.77 (3.44)	32.63 (3.32)	0.8662
Gamma (dB)	25.80 (2.99)	25.66 (3.91)	26.86 (4.86)	0.8574

**Table 3:** Mean (standard deviation) global relative power in subgroups. Bold indicates statistically significant differences.

	PD-CogN	PD-MCI	PD-D	*p*
Delta (%)	12.38 (1.64)	12.69 (2.18)	12.96 (1.72)	0.4531
Theta (%)	12.62 (1.64)	13.36 (2.32)	13.76 (2.14)	0.0656
Alpha (%)	15.16 (1.43)	15.22 (1.30)	14.80 (1.55)	0.5107
Low Alpha (%)	6.51 (0.81)	6.57 (0.77)	6.39 (0.78)	0.7040
High Alpha (%)	8.65 (0.78)	8.65 (0.74)	8.41 (0.80)	0.4210
**Beta (%)**	**37.69 (2.06)**	**37.32 (2.61)**	**36.27 (1.93)**	**0.0413**
Gamma (%)	22.15 (3.00)	21.41 (3.65)	22.22 (4.1)	0.6117
**alpha/theta ratio**	**1.21 (0.13)**	**1.17 (0.18)**	**1.09 (0.13)**	**0.0040**
**SPR**	**2.15 (0.31)**	**2.09 (0.43)**	**1.96 (0.34)**	**0.0385**

**Table 4:** The advantages and drawbacks of the proposed approach and other studies in the field.

	Advantages	Drawbacks
Proposed work	comprehensive neuropsychological asessments data collected during routine diagnostics relatively large sample group of 98 subjects 3 subgroups: PD-CogN (*n* = 43), PD-MCI (*n* = 30) and PD-D (*n* = 25)	all patients were treated with dopaminergic therapy, which might potentially influence the background activity in PD patients [60] no long-term follow up
Cozac et al., 2016 [48]	comprehensive neuropsychological and psychiatric asessments fully automated processing of high-resolution EEG	short mean observation period of 3 years relatively small sample size
Caviness et al., 2007 [29]	3 subgroups: PD-CogN, PD-MCI, PD-D (total *n* = 80)	earlier, different from the current criteria for the diagnosis of PD-MCI
Caviness et al., 2015 [38]	71 PD subjects with a mean follow-up of 3.9 years	subjects were part of an ongoing brain bank cohort, there may have been a bias introduced
Moritta et al., 2011 [45]	3 subgroups PD-CogN, PD-MCI, PD-D	earlier, different from the current criteria for the diagnosis of PD-MCI
Bousleimann et al., 2014 [32]	2 subgroups PD-CogN and PD-MCI (*n* = 53)	relatively limited sample size
Gu et al., 2016 [31]	2 subgroups PD-MCI (*n* = 17) and PD-D (*n* = 9)	short mean observation period of 2 years relatively small sample size

## Data Availability

The data presented in this study are available on request from the corresponding author.

## Abbreviations

The following abbreviations are used in this manuscript:

PD	Parkinson’s disease
MDS	International Parkinson and Movement Disorder Society
PD-CogN	Normal cognition
PD-MCI	Mild cognitive impairment
PD-D	Dementia
EEG	Electroencephalogram
QEEG	Quantitative EEG
H&Y	Hoehn and Yahr Scale
UPDRS	Unified Parkinson Disease Rating Scale
LED	Levodopa Equivalent Dose
MMSE	Mini-Mental State Examination
MoCA	Montreal Cognitive Assessment
CDT	Clock drawing test
ACE III	Addenbrooke’s Cognitive Examination III
BVRT	Benton Visual Retention Test
BDI	Beck Depression Inventory
PSD	Power spectral density
SPR	Spectral Power Ratio
